# Faculty versus students: different perceptions of misconducts at university

**DOI:** 10.3389/fpsyg.2024.1348057

**Published:** 2024-02-20

**Authors:** Nuria Alcalde-Fradejas, Mercedes Marzo-Navarro, Marisa Ramírez-Alesón

**Affiliations:** ^1^Department of Management and Business Organization, University of Zaragoza, Zaragoza, Spain; ^2^Department of Marketing Management and Market Research, University of Zaragoza, Zaragoza, Spain

**Keywords:** academic integrity, survey, economics and business school, student, teacher

## Abstract

**Introduction:**

Academic integrity is a goal to be achieved by university institutions, and student academic behavioral misconduct is a phenomenon to be fought and eradicated. Two of the main problems faced by universities in this area are: (1) the lack of consensus among students and faculty on the seriousness of acts considered academic misconduct; and (2) the difficulty of noticing and controlling certain student behaviors. The main aim of this paper is to assess the importance of these two problems.

**Methods:**

For this purpose, the authors compare, on the one hand, students’ and teachers’ perceptions of the seriousness of different types of dishonest and inappropriate behaviors and, on the other hand, the frequency with which they report that these misconducts occur. Two samples were taken from the responses of students and teachers of the Economics and Business School of the University of Zaragoza. The first consisted of 333 students and the second of 72 teachers. The academic misconducts asked about were grouped into three categories: academic works, exams, and interpersonal relationships in the classroom. Nonparametric tests were used to study the significance of the differences observed in the responses of students and teachers.

**Results:**

Results show that the greatest differences in the assessment of the seriousness of academic misconducts are in the group referring to interpersonal relationships. In terms of frequency, the study reveals that there is a serious problem of moral hazard in some of the behaviors analyzed, since the frequency with which teachers notice these practices is lower than that expressed by students.

**Discussion:**

Based on these results, possible measures to be adopted in universities in order to eradicate the academic misconduct problem are discussed.

## 1 Introduction

One of the objectives of university institutions is to train future highly qualified professionals. The fulfilment of this purpose entails two important aspects (Cuadrado et al., 2019; Muhammad et al., 2020). Firstly, universities seek to teach technical and professional knowledge of the highest level to the new generation. To achieve this, they must provide their students with the best tools to reach their academic potential and excel. Secondly, these institutions also have the responsibility to strengthen the values, principles, and moral development of their students. This means promoting the comprehensive education of students, not only in terms of specialized professional knowledge, but also instilling in them a strong foundation of ethical values that foster their personal growth. Academic integrity is essential for education excellence (Sbaffi and Zhao, 2022).

By providing quality education, universities empower their students. This not only benefits their professional future, but also contributes to the creation of a better society. In order to provide quality, student-centered training, it is necessary to develop curricula and use teaching methods that meet the diverse needs of the economy and the labor market in a global context. However, universities cannot be solely responsible for this, as the teaching-learning process involves two main actors: teachers and students. The former are responsible for teaching, while the latter are responsible for the learning process. A successful teaching-learning process requires both agents to perform their tasks correctly.

When considering students, it is observed that some behave dishonestly in order to achieve better results or to achieve them more quickly. Thus, the eradication of academic dishonesty is currently one of the most important concerns of universities (Singh et al., 2016), since dishonest behavior in the academic sphere implies a lack of ethics and morals that may affect not only the future professional behavior of current students, but also their own personal development. Additionally, it is important to monitor other misconducts that may not necessarily be aimed at achieving better academic results, but are still considered inappropriate or unacceptable within the established code or guidelines of conduct of a particular group, profession, or organization. For instance, students’ uncivil behaviors in a classroom that significantly disrupt the learning process would contradict the values of respect and responsibility that are expected of them. According to Brooks et al. (2011), there is a correlation between the student’s behavior regarding academic integrity and classroom civility, as both involve the student’s willingness to adhere to the rules and regulations of the university.

From a social perspective, if academic misconduct is not corrected by university institutions, there is a risk that students will turn these misbehaviors into normal behaviors and bring them to their personal and professional life once they have finished their studies (Guerra Torrealba, 2017). In this regard, the study by Fida et al. (2018) examined the relationship between self-efficacy, moral detachment, and academic dishonesty, concluding that there is a potential risk of individuals entering a vicious cycle in which academic dishonesty becomes a cognitive process. This means that they may develop the belief that dishonesty is natural and find more reasons and efficient ways to repeat it.

In addition, the concern about eradicating academic misconducts is also based on its impact on the very prestige and reputation of universities (Luck et al., 2022). Students who cheat will become ill-prepared graduates who will enter the job market, resulting in underachieving or inappropriately skilled employees. Employers may then attribute their underperformance to inadequate training, thereby devaluing the reputation of the institutions where the academic dishonesty has occurred (Barbaranelli et al., 2018; Bashir and Bala, 2018; Lord Ferguson et al., 2022; Malesky et al., 2022).

Taking all these points into consideration, it becomes necessary for academic institutions to include the achievement of academic integrity among their goals (Cebrián-Robles et al., 2018), analyzing the causes and seeking the most appropriate solutions. Nevertheless, there are two main problems that universities face when dealing with academic misconduct. The first one is the absence of a clear consensus on the acts that constitute this behavior and their seriousness. The second one is the difficulty of noticing and monitoring certain students’ misconducts.

Regarding the first problem, there is a lack of agreement among the different actors that make up the university (Lord Ferguson et al., 2022), since students, teachers and managers do not entirely agree on the identification of a dishonest behavior (Gullifer and Tyson, 2014; Waltzer and Dahl, 2023). Also, on many occasions each university has its own integrity standards, which often vary among universities making it difficult to have a generally accepted concept (Błachnio et al., 2022).

The second problem refers to the faculty’s difficulties in noticing, controlling and sanctioning acts of academic misconduct. This is known as moral hazard problem. Moral hazard occurs in contexts of asymmetric information, when one party has information about its behavior (in our case students) that the other party (teachers) cannot obtain or monitor, and yet, it is affected by its consequences. This may cause those who have the information to engage in inappropriate behavior or take advantage of certain circumstances knowing that the cost of the consequences will be borne by others. This difficulty has been greatly aggravated by the development of information and communication technologies (ICT). They facilitate traditional dishonest behaviors (e.g., copying during exams and plagiarizing academic work) but also favor the emergence of new ones (Meiring, 2019). While there are programs to detect cyberplagiarism, there is also software designed for paraphrasing (Birks et al., 2020). Moreover, during the recent Covid-19 pandemic, the teaching-learning process was completely developed online, so students had more opportunities to cheat on online assessments according to Wiley (2020). Chirumamilla et al. (2020) and Reedy et al. (2021) confirm that there is a greater potential risk of fraudulent behavior during non-face-to-face exams.

In order to shed light on the problem of academic misconduct in universities, this paper pursues a twofold objective. The first one is to analyze and compare the perception of teachers and students of the seriousness of different types of academic misconducts. The study includes dishonest behaviors related to the production of works, dishonest behaviors in exams, and inappropriate behaviors related to everyday aspects of respect and coexistence. The second one is to identify and compare the frequency of such behaviors, according to students and teachers. For this purpose, a sample of students and a sample of teachers from the Economics and Business School of the University of Zaragoza were used.

This work is pioneering not only because it incorporates the teacher’s perspective, which has hardly been addressed (some examples are the works of Sureda et al., 2009; Stevens, 2013; Blau et al., 2021) but also because it deals with this problem from a dual perspective: teacher versus student (e.g., Marcano et al., 2023; Rettinger, 2023). It also encompasses misconducts not only in the writing of exams (as analyzed by Amzalag et al., 2022), but also in the production of academic works, as well as other instances of student misbehavior in university life. The results also make it possible to identify dishonest and inappropriate behaviors and to assess their seriousness and frequency. This helps to delimit the problem, which is essential for developing effective and efficient measures to prevent such behaviors. Therefore, this work can serve as a basis to guide the heads of academic institutions in the establishment of measures in their university centers.

## 2 Theoretical background

### 2.1 Academic integrity

Academic integrity refers to trustworthy, respectful, fair, and responsible behavior (Sefcik et al., 2020). It is also one of the main ways to ensure quality education, a primary goal of universities (Ozoliņa and Bēriņa, 2021). The opposite concept is academic dishonesty, which encompasses various offenses (Etgar et al., 2019). The conceptualization of this misconduct is complex and has evolved over time, as discussed in the review by Ramos et al. (2020), as it is influenced by historical and social contexts. In addition, the lack of consensus among the involved parties (university managers, teachers, and students, among others) contributes to further confusion regarding this concept (Lord Ferguson et al., 2022). For instance, each university has its own regulations regarding codes of conduct or ethical codes. Muñoz-Cantero et al. (2019) state that the lack of agreement on the definition of the concept is due to “*its universality, multidimensionality, multicausality, and cultural determinants*.”

While there are many definitions, academic dishonesty can be described as any intentional behavior by students during their teaching-learning process that breaks the established norms or ethical principles of the educational institution and, in addition, gives them an unfair or undeserved advantage over the rest of their peers (Reyneke et al., 2021).

Academic integrity, however, is more than the absence of cheating (Christensen Hughes and Eaton, 2022). Uncivil conduct in class that significantly disrupts the learning process or any other misconduct that could affect everyday aspects of respect and coexistence and contravenes the values on which academic integrity is based should be considered inappropriate or unacceptable behavior. It is important to maintain a respectful and cooperative environment. Therefore, universities should be concerned about such behavior. As suggested by Brooks et al. (2011) student behavior regarding academic integrity and classroom civility are linked, as both refer to the student’s willingness to respect the rules and regulations of the university.

### 2.2 Academic misconducts

There is no homogeneity in the academic misconducts studied in the literature to date. Additionally, extensive research on this topic reveals numerous and diverse academic misconducts. These can be influenced by various cultural, sociodemographic, contextual, and temporal factors, making it challenging to determine students’ academic misconducts. Furthermore, not all of these behaviors are recognized as equally serious, making them difficult to identify (Ramos et al., 2020).

There are two main criteria for grouping the behaviors considered academic misconducts. Firstly, these behaviors can be categorized under different types of academic integrity violations, such as cheating, plagiarism, fabrication, and facilitation (e.g., Pavela, 1997; Blau et al., 2021). This classification belongs to the field of ethics, which focuses on what is right and what is wrong.

Cheating is the intentional use of study materials, information, or any type of aid that is prohibited, including consultation with others. Plagiarism (either partial or total) is a literary theft that consists of using any type of text, image, figure, or table that has been prepared by others and presenting it as one’s own without citing the source. It is one of the most frequently analyzed dishonest behaviors and the results show not only the high prevalence of plagiarism among university students (Javaeed et al., 2019; Curtis and Tremayne, 2021; Hopp and Speil, 2021), but also where students and teachers agree that it is dishonest behavior (Denney et al., 2021). Fabrication is the creation of information or data that do not exist. Finally, facilitation is helping others to engage in any kind of dishonest behavior. There is general agreement in the literature that all of these behaviors are dishonest.

Secondly, behaviors can be grouped according to the tasks in which they occur (Comas et al., 2011; Sureda-Negre et al., 2016; Navarro et al., 2023). In particular, Comas et al. (2011) differentiate between: (i) behaviors related to the preparation and presentation of academic works, (ii) behaviors related to exams, and (iii) behaviors in the context of interpersonal relationships and those related to everyday aspects of respect and coexistence.

The first group, behaviors related to the preparation and presentation of academic works, includes dishonest behaviors such as copying ideas or fragments of text without citing the corresponding source in the bibliography (e.g., Bashir and Bala, 2018; Denney et al., 2021), presenting an assignment prepared by another person as one’s own work (e.g., Comas et al., 2011; Cebrián-Robles et al., 2018) or forging the bibliography and resources consulted to write an academic assignment (e.g., Bashir and Bala, 2018; Sureda-Negre et al., 2020).

Among the dishonest behaviors during exams, the following stand out: copying someone during the exam (e.g., Comas et al., 2011; Bashir and Bala, 2018), accessing unauthorized information during an exam either through the use of traditional “crib sheets” or through technological means (mobile phone, etc.) (e.g., Comas et al., 2011; Bashir and Bala, 2018), allowing someone to copy you or obtaining detailed information about the content of the exam before taking it (e.g., Comas et al., 2011), among others.

The third group, behaviors in the context of interpersonal relationships and those related to everyday aspects of respect and coexistence, would include those behaviors that, although not related to cheating, contradict the values of respect and responsibility that are expected of students (Ochoa et al., 2023). Thus, these uncivil behaviors in the classroom should be eradicated because they are inappropriate. These include damaging equipment or furniture at academic facilities, damaging the equipment and personal belongings of other students, damaging the work or materials of other students, interfering with other students’ work or exams, frustrating their activities, and showing a lack of respect toward other students or staff, among others (e.g., Comas et al., 2011).

As reflected in the literature review by Newton (2018) and Awasthi (2019), the first two groups (cheating on assessment works and exams) are the most visible manifestations of academic dishonesty among the student body in university systems around the world. In addition, they are universally considered as unlawful conducts, while the behaviors included in the third group are simply considered to be inappropriate or reprehensible behaviors.

### 2.3 The phenomenon of academic misconduct

Academic misconduct is not a recent problem, as it has been documented for a long time. In China, for example, people taking civil service examinations were searched for “impermissible material” that would help them copy during the tests (Brickman, 1961). Cizek (1999) reports similar incidents in countries such as Saudi Arabia, Israel, India, South Africa, Nigeria, Brazil, Ireland, Cuba, England, and Mexico. Also, in the United States, this topic has been treated in the academic literature and investigated by educational institutions since the first half of the last century (Yepsen, 1927; Corey, 1937; Drake, 1941).

During the present century, multiple studies addressing the problem can be found anywhere in the world (Barbaranelli et al., 2018; Bashir and Bala, 2018; Sureda-Negre et al., 2020). Academic dishonesty has been, and continues to be, a global problem (see Marques et al., 2019), even an epidemic (Vaamonde and Omar, 2008), with prevalence increasing in recent years (e.g., Grira and Jaeck, 2019; Birks et al., 2020; Harper et al., 2021). Research has demonstrated that academic misconduct is a common behavior among students (Krou et al., 2021; Chiang et al., 2022; Christensen Hughes and Eaton, 2022; Zhang et al., 2022). Additionally, Peled et al. (2019) found that most students engage in academic misconduct at some point during their studies.

The problem of academic misconduct is also present in the Spanish context. 42% of university students admit to committing academic offenses when writing academic papers combining their own content with fragments of text from the Internet, and most of them (62%) consider this to be a common practice among the rest of the university population (Comas et al., 2011). This gives an idea of the concern of institutions and society about this situation.

However, according to Bergadaà (2020), there is not a clear solution to this issue, and further research is necessary. Therefore, our work aims to answer three main research questions based on previous literature:

1. What is the severity of different types of dishonest and inappropriate behaviors as perceived by students and teachers?
2. How often do students and teachers perceive these misconducts occur?
3. Do teachers and students perceive the severity and frequency of different types of dishonest and inappropriate behaviors differently?

## 3 Materials and methods

A quantitative study based on information collected through two surveys, one for students and one for teachers, was chosen to achieve the research objectives. Non-parametric statistical tests were used to analyze the significance of the differences found.

The two surveys that constitute the source of information for this paper were very similar in content (which facilitates their comparison), were distributed online (using Google Doc forms), and were anonymous (to ensure respondent confidentiality). The first one, aimed at students enrolled in undergraduate studies at the Economics and Business School of the University of Zaragoza, was conducted between January and March 2020. The second one, addressed to the teaching staff of undergraduate studies at the Economics and Business School of the University of Zaragoza, was conducted between February and March 2023. In both cases, the questionnaire was sent to the institutional e-mail accounts of students and teachers with the permission of the university authorities.

### 3.1 Sample

The responses to both surveys provide two samples: one that collects the assessment of dishonest behaviors and the frequency with which they occur from the students’ perspective, and another that collects the same aspects, but from the teachers’ point of view.

The student population under study corresponds to 3,869 students enrolled during the 2019/20 academic year in any of the undergraduate degrees offered by the Economics and Business School of the University of Zaragoza. We focus on economics and business undergraduate students because previous literature suggests that their ethical level is lower than that of other students (McCabe et al., 2006; Lord Ferguson et al., 2022). A final sample of 333 valid questionnaires is obtained (response rate close to 9%; for a confidence level of 95%, the error is 5.1%). Most individuals are 22 years old or younger and female. A full description of the sample is provided in Table 1.

**Table 1:** Description of the samples.

	Teachers	%		Students	%
Age	> 60 years	10.4	Age	≤22 years	66.3
	51–60 years	44.8		> 22 years	33.7
	41–50 years	20.9			
	31–40 years	20.9			
	≥ 30 years	3.0			
Gender	Female	51.3	Gender	Female	60.4
	Male	49.3		Male	39.0
Year of study	First	26.8	Year of study	First	15.6
	Second	36.6		Second	18.4
	Third	23.9		Third	26.2
	Fourth	12.7		Fourth	39.9
Professional category	Professors	12.5			
	Associate professors	54.2			
	Contract teachers	13.9			
	Trainee teachers	5.6			
	Part-time teachers & other	13.9			

For the faculty sample, the study population is made up of 318 teachers who taught at the Economics and Business School of the University of Zaragoza during the 2022/23 academic year in any of its undergraduate degrees. The final sample consists of 72 valid questionnaires (response rate close to 23%; for a confidence level of 95%, the error is 10.2%). As Table 1 shows, the sample is almost equally composed of women and men. Most teachers are over 50 years, followed in importance by those between 31 and 40 years of age. Regarding the professional category, the sample is mainly composed of public servant teachers.

### 3.2 Instrument

Both the questionnaire for students and the one for teachers contain two blocks of questions. There is a first common block of questions on the items under study, based on previous literature and following the classification of dishonest behaviors by Comas et al. (2011). This classification is widely used and helps us to analyze behaviors that are not purely dishonest but may still affect appropriate interactions within university life. The second block includes the sociodemographic characteristics of the respondents.

In the first block, the student or the teacher is asked, on the one hand, to rate the level of appropriateness of a series of behaviors (21 items measured on a scale from 0, totally inappropriate to 10, totally appropriate), and, on the other hand, to indicate the observed frequency of these behaviors (from 0, never to 10, always) for the same 21 items. Specifically, there are seven items related to dishonest behaviors in the production of academic work; seven refer to dishonest behaviors during the writing of exams, and the last seven items are related to inappropriate behaviors in interpersonal relationships and everyday aspects of respect and coexistence. Table 2 shows the items that have been used and validated in previous studies.

**Table 2:** Items in the questionarie.

Ítem		Some previous literature
Works	W1-Including people who did not work on the list of authors of a project	Almeida et al. (2010) and Sureda-Negre et al. (2020)
	W2-Manipulating information from other studies to suit one’s own interests	Lambert et al. (2003), Comas et al. (2011), Bashir and Bala (2018), Lado and Varela Martínez (2019), and Sureda-Negre et al. (2020)
	W3-Copying another classmate’s exercise or work	Comas et al. (2011), Cebrián-Robles et al. (2018), and Reskala Sánchez (2020)
	W4-Copying books, magazines, websites without expressly quoting them	Comas et al. (2011), Dick et al. (2001), Denisova Schmidt (2017), Guerrero et al. (2017), Bashir and Bala (2018), Cebrián-Robles et al. (2018), Lado and Varela Martínez (2019), Reskala Sánchez (2020), and Sureda-Negre et al., 2020
	W5-Submitting a Project outside of the established deadline	Lambert et al. (2003), Bashir and Bala (2018), Reskala Sánchez (2020)
	W6-Not collaborating on the completion of a group project	Bretag et al. (2014), and Bashir and Bala (2018)
	W7-Buying a class Project, undergraduate disseartion	Dick et al. (2001), Lambert et al. (2003), Comas et al. (2011), Denisova Schmidt (2017), Bashir and Bala (2018), Lado and Varela Martínez (2019), and Sureda-Negre et al. (2020)
Exams	E1-Using technology copy during an exam	Comas et al. (2011), Guerrero et al. (2017), Bashir and Bala (2018), Lado and Varela Martínez (2019), and Sureda-Negre et al. (2020)
	E2-Using prohibited material during an exam (notes, cheat sheets, …)	Dick et al. (2001), Lambert et al. (2003), Comas et al. (2011), Denisova Schmidt (2017), Guerrero et al. (2017), Bashir and Bala (2018), Lado and Varela Martínez (2019), and Sureda-Negre et al. (2020)
	E3-Looking at another student’s exam answer	Lambert et al. (2003), Almeida et al. (2010), Comas et al. (2011), Denisova Schmidt (2017), Guerrero et al. (2017), Bashir and Bala (2018), Sureda-Negre et al. (2020), and Reskala Sánchez (2020)
	E4-Asking another student during the exam	Lado and Varela Martínez (2019) and Sureda-Negre et al. (2020)
	E5-Allowing a classmate to copy their exam	Almeida et al. (2010), Comas et al. (2011), Guerrero et al. (2017), Lado and Varela Martínez (2019), Sureda-Negre et al. (2020), and Reskala Sánchez (2020)
	E6-Having access to the exam in advance	Comas et al. (2011), Guerrero et al. (2017), Bashir and Bala (2018), Sureda-Negre et al. (2020), and Reskala Sánchez (2020)
	E7-Writing the exam for another student	Dick et al. (2001), Lambert et al. (2003), Comas et al. (2011), Guerrero et al. (2017), Lado and Varela Martínez (2019), Sureda-Negre et al. (2020), and Reskala Sánchez (2020)
Interpersonal relationships	IR1-Throwing garbage on the floor or leaving it on tables and seats	Suggested by the authors
	IR2-Property theft behavior	Suggested by the authors
	IR3-Damaging furniture	Comas et al. (2011)
	IR4-Interrupting or hindering the attention of the class	Comas et al. (2011)
	IR5-Entering the class late or leaving it early without a justified cause	Suggested by the authors
	IR6-Drinking and eating in class	Suggested by the authors
	IR7-Use of electronic devices in class for other activities not related to it	Suggested by the authors

The questionnaire was sent in advance to six selected experts, so that it was answered by experts in education, experts in all macro knowledge areas, representatives of universities and external professionals, maintaining the parity between men and women. A pre-test was also conducted to check their understanding of the questionnaire and whether there were any missing behaviors not initially included.

In order to validate the measurement scale used, we determined the underlying dimensional structure. For this purpose, we performed a principal component analysis with varimax rotation (IBM SPSS Statistics 26) for the 14 items proposed in order to measure the aspects related to dishonest behaviors. The results obtained show that there are two components that explain 49.6% of the variance. The first one is made up of seven items (E1 to E7) and includes aspects related to dishonest behavior in exams. This component explains 29.1% of the variance and Cronbach’s alpha coefficient for it is 0.9. The second identified component is made up of seven items (W1 to W7) and includes aspects regarding dishonest behaviors related to the preparation and presentation of works. It explains 20.5% of the variance and Cronbach’s alpha coefficient takes a value of 0.7.

Following the same procedure, we performed a principal component analysis for the seven items related to inappropriate behaviors in the context of interpersonal relationships and those related to everyday aspects of respect and coexistence. The results obtained show that there are two components that explain 51.1% of the variance. The first one is made up of three items (IR1 to IR3) and includes aspects related to the respect for university’s property or for that of the students themselves. It explains 26.5% of the variance and Cronbach’s alpha coefficient is 0.6. The second identified component is made up of four items (IR4 to IR7) and includes aspects related to the students’ attitude in the classroom. This component explains 24.6% of the variance and Cronbach’s alpha coefficient takes a value of 0.6.

## 4 Results

In order to determine how dishonest some of the students’ behaviors are and how often they are detected, we performed a descriptive analysis based on the calculation of the mean values obtained in each of the samples (students and teachers) for every item in each of the three groups of behaviors presented. We also wanted to analyze whether there were differences between the perceptions of students and teachers regarding the degree of inappropriateness of the behaviors and their frequency. To achieve this, we performed the statistic contrasts of mean differences. Since the samples do not follow a normal distribution, we used the Mann–Whitney U test statistic. We also applied Bonferroni correction for multiple hypotheses to control the family-wise error (the probability of falsely rejecting one or more hypotheses in a family of hypotheses).

Regarding dishonest behaviors related to the preparation and presentation of academic works, the results in Table 3 show that teachers consider all the behaviors proposed in the study to be highly inappropriate, since their mean values are below 3 (mean values between 0.74 and 2.70). This consensus on the inappropriateness of this type of behavior would lead one to expect that such behaviors would not be noticed in reality or that their frequency would be close to zero. However, it is not the case, with mean frequencies ranging from 2.02 to 5.66. Detailing the results, the faculty considers the purchase of a class work or a degree’s final project to be the most inappropriate behavior (0.74). Fortunately, however, it is the least reported form of dishonesty at university (2.02). On the contrary, certain dishonest behaviors are detected with some assiduity, with mean frequencies around 5, such as copying bibliographic material without citing the source (5.66), lack of equal collaboration in completing group work (5.18), trying to persuade the teacher to accept work after the established deadline (4.91) and copying work among students (4.53).

**Table 3:** Dishonest behaviors related to the preparation and presentation of works.

	Appropriateness			Frequency		
	Teacher	Student	Mann–Whitney’s	Teacher	Student	Mann–Whitney’s
	Average (s.d)	Average (s.d)		Average (s.d)	Average (s.d)	
Including people who did not work on the list of authors of a project	1.45 (1.782)	1.63 (2.074)	10901.0	4.06 (3.048)	4.37 (3.496)	9787.5
Manipulating information from other studies to suit one’s own interests	1.38 (1.885)	1.84 (2.064)	9493.5	4.08 (2.863)	3.25 (3.007)	8425.0
Copying another classmate’s exercise or work	1.19 (1.803)	3.45 (2.882)	5978.5***	4.53 (2.551)	5.74 (3.199)	7499.5***
Copying books, magazines, websites without expressly quoting them	1.75 (2.024)	2.32 (2.180)	9144.0	5.66 (2.835)	4.58 (3.238)	8065.5*
Submitting a Project outside of the established deadline	2.70 (2.060)	1.62 (2.363)	6808.5***	4.91 (3.049)	1.99 (2.761)	4899.5***
Not collaborating on the completion of a group project	2.35 (2.064)	1.04 (1.708)	6263.5***	5.18 (3.172)	5.97 (3.264)	8750.5
Buying a class Project, undergraduate disseartion	0.74 (1.492)	1.05 (2.070)	10387.0	2.02 (2.826)	1.27 (2.448)	8651.5

s.d.: Standard deviation. *** *p*-value with Bonferroni correction < 0.01; ** *p*-value with Bonferroni correction < 0.05; * *p*-value with Bonferroni correction < 0.1.

It is interesting to analyze whether the perceptions of teachers and students in relation to the appropriateness and frequency of these behaviors coincide or not. As shown in Table 3, there are statistically significant differences in the assessment of the appropriateness of behaviors in three of the seven items grouped under the dimension “Dishonest behaviors related to the preparation and presentation of works.” Thus, teachers consider it more inappropriate to copy the exercises or work of other classmates than students do. On the contrary, students perceive the lack of equal collaboration in completing group work and the attempt to persuade teachers to accept work after the deadline as more inappropriate behaviors than teachers do.

There were not only discrepancies between the opinions of teachers and students regarding the appropriateness of behaviors, but also statistically significant differences in the frequency of three of the seven items related to dishonest behaviors in the preparation and presentation of academic works. The analysis of these results reveals two clear patterns. On the one hand, students notice a higher frequency than teachers of the misbehavior that implies copying the work of other classmates. Teachers, on the other hand, believe that it is more common to copy sources without citation and to try to convince the teacher to accept works after the deadline as well as to purchase degree’s final projects or works.

Table 4 shows the results obtained in order to analyze students’ dishonest behaviors related to exams. The data reflect that teachers agree on the fact that these behaviors are highly inappropriate, as the mean rating for most of the items is around 1 (between 0.12 and 1.72). However, when analyzing the frequency with which teachers notice these behaviors, the mean scores in all items are higher than 0 (between 0.67 and 4.67), which again shows that the reality is far from the expected situation of honesty. The most frequently observed acts are those related to copying in the exam (with values close to the midpoint of the scale −5-): looking at another student’s exam answer, asking another student during the exam, allowing a classmate to copy off their exam. The least frequent behaviors are writing the exam for another student, and having access to the exam in advance, all of which have values below 1. This result can be seen as positive since these behaviors were considered the most inappropriate by the faculty. It should be noted, however, that some of these behaviors cannot be directly observed by teachers because they do not occur in the classroom (e.g., access to the exam in advance), which influences their reported frequency.

**Table 4:** Dishonest behaviors in the exams.

	Appropriateness			Frequency		
	Teacher	Student	Mann–Whitney’s	Teacher	Student	Mann–Whitney’s
	Average (s.d)	Average (s.d)		Average (s.d)	Average (s.d)	
Using technology copy during an exam	0.52 (1.167)	0.49 (1.257)	10676.5	2.26 (2.354)	4.07 (3.674)	7726.0**
Using prohibited material during an exam (notes, cheat sheets, …)	0.52 (1.099)	0.73 (1.647)	10618.0	3.39 (2.398)	4.85 (3.579)	8031.0**
Looking at another student’s exam answer	1.11 (1.437)	1.25 (1.934)	10436.5	4.67 (2.616)	5.51 (3.414)	8524.0
Asking another student during the exam	0.98 (1.283)	1.01 (1.757)	10095.5	4.26 (2.606)	5.38 (3.467)	8027.0*
Allowing a classmate to copy their exam	1.72 (1.873)	1.68 (2.205)	10356.5	4.11 (2.735)	4.89 (3.422)	8790.0
Having access to the exam in advance	0.32 (0.963)	1.37 (2.412)	8530.0***	0.77 (1.935)	1.21 (2.374)	9352.0
Writing the exam for another student	0.12 (0.448)	0.40 (1.262)	10144.5	0.67 (1.861)	0.34 (1.342)	8846.0*

s.d.: Standard deviation. *** *p*-value with Bonferroni correction < 0.01; ** *p*-value with Bonferroni correction < 0.05; * *p*-value with Bonferroni correction < 0.1.

Comparing the students’ responses, Table 4 shows that most teachers and students agree on the fact that all the items grouped under the dimension “Dishonest behavior in the exams” are very inappropriate. Moreover, there is only a statistically significant difference in the assessment of the item “Having access to the exam in advance,” since even though students consider this behavior very inappropriate, they do so to a lesser extent than teachers do (0.32 for the teachers versus 1.37 for the students).

This consensus between students and teachers disappears when the average frequency with which these behaviors are noticed is analyzed. A noteworthy result is that in six of the seven items, the frequency observed by the students is higher than that observed by the teachers, and it is statistically significant in three of these behaviors: using technological means to copy; using unauthorized materials, and asking another student the answer during the exam. The only behavior teachers notice more than students (showing a statistically significant difference) is writing an exam for another student. However, its frequency in both cases is less than 1.

Table 5 shows the results obtained in order to analyze the situation of inappropriate behaviors in the context of interpersonal relationships and those related to everyday aspects of respect and coexistence. Although there is consensus among teachers that most of the behaviors analyzed are highly inappropriate, for the first time there are two behaviors with mean values above 3: drinking and eating in class (3.39) as well as entering the class late or leaving it early without a justified cause (3.27). Both behaviors score higher because they are not really dishonest behaviors, although they may be inappropriate because they could be detrimental to the smooth running of the class.

**Table 5:** Inappropriate behaviors in the context of interpersonal relationships and those related to everyday aspects of respect and coexistence.

	Appropriateness			Frequency		
	Teacher	Student	Mann–Whitney’s	Teacher	Student	Mann–Whitney’s
	Average (s.d)	Average (s.d)		Average (s.d)	Average (s.d)	
Throwing garbage on the floor or leaving it on tables and seats	0.97 (1.347)	0.31 (0.779)	7373.5***	3.76 (2.632)	3.73 (3.176)	10307.5
Property theft behavior	0.23 (0.740)	0.24 (1.027)	10603.5	0.55 (1.541)	0.76 (1.596)	9715.0
Damaging furniture	0.67 (1.128)	0.47 (1.055)	9457.0*	2.27 (2.574)	3.34 (3.048)	8,638
Interrupting or hindering the attention of the class	1.99 (2.332)	1.22 (1.622)	8796.5**	6.88 (2.471)	5.47 (3.306)	8024.5**
Entering the class late or leaving it early without a justified cause	3.27 (2.660)	3.56 (3.123)	10854.0	6.69 (2.443)	6.09 (3.159)	9425.5
Drinking and eating in class	3.39 (2.348)	4.31 (2.730)	8759.0**	5.40 (2.764)	6.10 (2.712)	9045.0
Use of electronic devices in class for other activities not related to it	2.46 (2.888)	3.74 (2.818)	7822.5***	7.30 (2.236)	6.62 (2.595)	8895.0

s.d.: Standard deviation. *** *p*-value with Bonferroni correction < 0.01; ** *p*-value with Bonferroni correction < 0.05; * *p*-value with Bonferroni correction < 0.1.

When analyzing the frequencies, it is observed that there are behaviors with mean frequency values higher than 5 (half of the scale): using electronic devices in class for other activities not related to it (7.30), interrupting or hindering the attention of the class (6.88), entering/leaving class late/early without a justified cause (6.69). These results reflect the existence of a problem that could be affecting the development of teaching and classroom performance. Property theft behavior, namely a crime, is the least frequent one. However, since its average value is greater than 0, it is a problem that should be eradicated.

Table 5 shows statistically significant differences in five of the seven items grouped under the dimension “Inappropriate behaviors in the context of interpersonal relationships and those related to everyday aspects of respect and coexistence.” It highlights that students find drinking or eating during class, or using electronic devices for other activities not related to it less inappropriate than teachers do. Also, teachers value throwing garbage on the floor or leaving it on tables and seats, damaging furniture, and interrupting class as more inappropriate behaviors than students do (in a statistically significant way) although the values are always below 2 for both samples.

However, there is greater consensus between teachers and students when frequency is analyzed, since statistically significant differences are obtained in only one item. Specifically, interruptions in class are noticed more frequently by teachers, although it should be noted that the mean frequency values are higher than 5 for both samples.

Finally, once the item-by-item analysis was completed, we analyzed whether there were any significant differences between the degree or level of inappropriateness that students and teachers assign to two sets of behaviors. These were, on the one hand, dishonest behaviors in works and exams and, on the other hand, inappropriate behaviors in the context of interpersonal relationships and those related to everyday aspects of respect and coexistence.

In order to do this, we calculated the average rating assigned to the items included in the first two blocks (dishonest behaviors in works and exams) and that of the items included in the third block (inappropriate behaviors) for each individual (teacher or student). We then analyzed whether there were any statistically significant differences. Results are shown in Table 6.

**Table 6:** Differences between dishonest and innappropriate behaviors.

	Teacher	Student	Differences of means Mann–Whitney U test
	Average (s.d)	Average (s.d)	
Dishonest behaviors in works and exams	1.19 (1.094)	1.41 (1.182)	9283.5
Innapropriate behaviors	1.86 (1.484)	1.98 (1.066)	9298.5*
Differences of means Wilcoxon signed rank test (*Z*-value)	4.928***	8.936***	

s.d.: Standard deviation. *** *p*-value < 0.01; ** *p*-value < 0.05; * *p*-value < 0.1.

As shown in Table 6 in the case of teachers, the items included in dishonest behaviors in works and exams obtain an average rating of 1.19, while the ones included in the block of inappropriate behaviors in the context of interpersonal relationships and those related to everyday aspects of respect and coexistence reach an average rating of 1.86. If we apply the Wilcoxon signed rank test (a non-parametric statistical test used to compare two dependent samples), the differences are significant at 1%. This suggests that, as expected, teachers consider dishonest behaviors in the completion of works and exams to be significantly more serious than inappropriate behaviors in the context of interpersonal relationships and those related to everyday aspects of respect and coexistence.

In the case of students, the average rating for the items related to dishonest behaviors in works and exams is 1.41, while the average value they assign to the set of inappropriate behaviors in the context of interpersonal relationships and those related to everyday aspects of respect and coexistence is 1.98. The differences are also statistically significant, confirming that students, like teachers, perceive dishonest behavior in exams and papers as significantly more serious.

Finally, we applied the Mann–Whitney U test to compare the ratings of students and teachers among themselves. It can be observed (last column of Table 6) that there are no statistically significant differences in the level of severity with which students and teachers perceive dishonest behaviors in the completion of works and exams. In the case of inappropriate behaviors in the context of interpersonal relationships and those related to everyday aspects of respect and coexistence, the differences are marginally significant at 10%, with teachers perceiving these behaviors as more serious than students.

## 5 Discussion and conclusions

Academic integrity is the expectation that all members of the academic community: teachers, students, researchers, and administrators act with honesty, trust, fairness, respect, and responsibility in all aspects of scholarly activity (Tertiary Education Quality and Standards Agency, 2022). It has become a top priority for universities (Ordóñez et al., 2021). Breaching academic integrity is also known as “academic misconduct” or “academic dishonesty.” Although academic dishonesty is not new, it seems to follow a growing trend, favored by the emergence of new tools (such as ICT, artificial intelligence, etc.). This phenomenon must be fought and eradicated (Yang et al., 2013; Tabsh et al., 2017).

This paper focuses on dishonest and inappropriate practices by university students in the academic environment. The literature review conducted in the works of Eaton and Edino (2018), Newton (2018), and Awasthi (2019) provides evidence that plagiarism and cheating on exams and assessment papers are the most visible manifestations of academic dishonesty among students in university systems worldwide. Furthermore, behaviors that are inappropriate in the context of interpersonal relationships and coexistence, even if they cannot be classified as dishonest, contradict the values of respect and responsibility that are expected of students (Ochoa et al., 2023).

Therefore, in this paper, we classified the dishonest and inappropriate behaviors according to the tasks in which they occur (Comas et al., 2011): (i) behaviors related to the preparation and presentation of academic works, (ii) behaviors related to exams and (iii) behaviors in the context of interpersonal relationships and those related to everyday aspects of respect and coexistence. We analyze a set of behaviors classified into these three groups, checking whether their seriousness is perceived, and how frequently these occur at the university. All this is performed under a double perspective, that of the student and that of the teacher, trying to analyze, as Amzalag et al. (2022) say the two sides of the same coin.

There are three main results regarding the perceived seriousness of the analyzed behaviors. In the first place, all these misconducts, whether they take place in the preparation of works, in exams or are related to the environment, were considered either dishonest or inappropriate and recognized as such by both teachers and students. This would reflect that students are really informed and know how to discern between right and wrong. However, some studies (e.g., McTernan et al., 2014) suggest that the decision-making process in academic misbehavior is not fully rational. This would mean that, even though students are informed about what is right and wrong at the description level, the decision to engage in those behaviors may be the result of an automatic process rather than a rational one. Secondly, teachers and students consider that the most serious form of academic misconduct is cheating on exams and works, while inappropriate behaviors in the context of interpersonal relationships and those related to everyday aspects of respect and coexistence are considered less serious. Finally, although students acknowledge the analyzed behaviors as inappropriate, it is noticed that they tend to assess them as less serious than teachers do.

Regarding the frequency with which these results are observed, there are three main findings worth noting. The first one is that all of the behaviors analyzed have been noticed at some point. This means that some of the students have a problem of academic dishonesty or engage in inappropriate behaviors, so it is necessary to take measures to discourage them from doing so. These results are comparable to those obtained in various countries, including previous research conducted in Spain (Ma et al., 2013; Hu and Lei, 2015; Comas-Forgas and Sureda-Negre, 2016; Curtis and Clare, 2017; Marzo-Navarro and Ramírez-Alesón, 2023). The second finding is the fact that students notice more than half of the analyzed behaviors more often than teachers, especially those related to exams. This would show that teachers are not always aware of these behaviors, as they are difficult to notice, since they occur in a context of asymmetric information between teachers and students, thus giving rise to a moral hazard problem. The student’s awareness of the teacher’s inability to prove inappropriate behavior may lead the student to act inappropriately without fear of punishment. Furthermore, certain behaviors such as “Having access to the exam in advance” or “Copying another classmate’s exercise or work” cannot be directly observed by teachers as they occur outside the classroom. This explains the differences in the perceived frequency between students and teachers. Finally, it is worth highlighting those dishonest behaviors that were found to be more frequent in our study, since vigilance should be intensified, and effective and efficient measures should be designed to eradicate them. In particular, the most common behaviors in the preparation of academic works are copying another classmate’s exercise, the copying of sources without citation, and not collaborating on the completion of a group project. Very frequent behaviors in exams are those related to all forms of copying during the exam (technological devices, looking at the answer, asking or allowing oneself to be copied). In the context of interpersonal relationships and everyday aspects, using electronic devices in class for activities not related to it, as well as interrupting and entering class late stand out due to their frequency. Therefore, universities could consider drafting rules of conduct and civility in the classroom as a measure to reduce this problem.

In light of these results and given the harmful effects of academic dishonesty, there are some recommendations for university institutions. Academic institutions should continue to try to positively influence student development and encourage students to become more ethical and honest individuals. A combination of preventive and corrective measures is needed to accomplish this task. Academic integrity education has beneficial effects for the student, the university institution that trains them as professionals and the society in which they will live and work, promoting a more sustainable and equitable future. Along these lines, some authors (e.g., Guerrero-Dib et al., 2020) suggest that educational institutions should provide ethical education to their students as a preventive measure against unethical practices. In addition, corrective measures should be implemented to detect and control such practices. Regarding faculty, universities need to ensure that their faculty have the appropriate knowledge, skills and tools to deal with these dishonest behaviors as they occur (Marsh and Campion, 2018; Newton, 2018). It is also crucial for universities to disseminate and enforce their disciplinary regulations so that students understand the consequences of such actions.

The main current study’s limitation is in the sample: teachers and students from the Economics and Business School of the University of Zaragoza. Therefore, the results cannot be generalized to other degree programs outside of business and economics. In this sense, McCabe et al. (2006) reported that business students cheat more than non-business students. Moreover, our results cannot be extrapolated to other contexts, given that although misconduct remains a global phenomenon, there are differences in misconduct rates across countries (Grira and Jaeck, 2019).

Further research on this topic is therefore needed, involving larger sample sizes and including not only grades from the economic field, but also from all other fields (e.g., scientific, medical, art,…). Additionally, it is still needed to investigate this problem in depth and to advance in the knowledge of the possible reasons for these undesired behaviors. Future research should explore the influence of specific preventive, control, and punitive measures on the perception of the seriousness and frequency of dishonest or inappropriate acts committed by students.

## Data availability statement

The raw data supporting the conclusions of this article will be made available by the authors, without undue reservation.

## Ethics statement

Ethical approval was not required for the studies involving humans because data was collected via a questionnaire. The studies were conducted in accordance with the local legislation and institutional requirements. Written informed consent for participation was not required from the participants as completion of the questionnaire implies consent.

## Author contributions

NA-F: Data curation, Data analysis, Methodology, Writing – original draft, Writing – review & editing. MM-N: Data curation, Data analysis, Methodology, Writing – original draft, Writing – review & editing. MR-A: Data curation, Data analysis, Methodology, Writing – original draft, Writing – review & editing.
